# LncRNA HOTAIR Promotes Proliferation of Malignant Melanoma Cells through NF-ϰB Pathway

**DOI:** 10.18502/ijph.v49i10.4696

**Published:** 2020-10

**Authors:** Jun WANG, Jingxin CHEN, Gang JING, Daoquan DONG

**Affiliations:** 1. Department of Burn and Skin Repair Surgery, Hainan General Hospital, Haikou, China; 2. Department of Burn and Skin Repair Surgery, Hainan Affiliated Hospital of Hainan Medical University, Haikou, China; 3. Department of Oraland Maxillofacial Surgery, Hainan General Hospital, Haikou, China; 4. Department of Oraland Maxillofacial Surgery, Hainan Affiliated Hospital of Hainan Medical University, Haikou, China

**Keywords:** HOTAIR long non-coding RNA, Malignant melanoma cells, NF-kappa B pathway

## Abstract

**Background:**

To study the effects of long non-coding ribonucleic acid (lncRNA) HOX transcript antisense intergenic RNA (HOTAIR) on the proliferation and apoptosis of malignant melanoma cells, and to explore its specific regulatory mechanism through the nuclear factor-kappa B (NF-ϰB) signaling pathway.

**Methods:**

LncRNA HOTAIR small-interfering RNAs (siRNAs) were designed and synthesized, and the effects of si-HOTAIR transfection on the proliferation and apoptosis of malignant melanoma cells were detected via cell counting kit-8 (CCK-8) assay, 4’,6-diamidino-2-phenylindole (DAPI) staining assay and flow cytometry, respectively. The gene expressions were determined using quantitative reverse transcription-polymerase chain reaction (qRT-PCR), the changes in NF-ϰB pathway-related proteins and apoptosis-associated proteins after interference in lncRNA HOTAIR were detected via Western blotting, and the level of NF-ϰB in each group was determined via ELISA.

**Results:**

The results of CCK-8 assay revealed that the cell proliferation rate significantly declined gradually in si-HOTAIR group compared with that in si-NC group and control group (*P*<0.05). The results of Western blotting and ELISA showed that the activity of NF-ϰB in si-HOTAIR group was weakened (*P*<0.05), suggesting that down-regulation of HOTAIR can suppress the activity of NF-ϰB. Compared with si-NC group and control group, si-HOTAIR group had remarkably increased gene and protein expressions of pro-apoptotic Bax, and remarkably decreased gene and protein expressions of anti-apoptotic Bcl-2 (*P*<0.05), demonstrating that down-regulation of HOTAIR can promote apoptosis.

**Conclusion:**

Down-regulation of lncRNA HOTAIR can inhibit the proliferation and promote the apoptosis of malignant melanoma cells and suppress the NF-ϰB pathway.

## Introduction

Melanoma is a malignant tumor derived from melanocytes, and it generally occurs in the skin where melanocytes are located, which is the most dangerous skin cancer (1, 2). In addition to over-exposure to ultraviolet rays, melanoma may be caused by genetic factors (3). Melanoma is characterized by a high proliferation rate and a high risk of metastasis, which can be usually cured in the early stage but is difficult to be treated once metastasis occurs (4). In recent years, some exciting progress has been made in the treatment of melanoma, but the morbidity rate of melanoma is increasing every year, so it is urgent to find effective treatment methods.

Long non-coding ribonucleic acids (lncRNAs) are transcribed from mammalian genomes, which are widely involved in regulating various diseases including cancer, thereby attracting attention in recent years (5). It is reported that the abnormal expression of lncRNAs has significant correlations with many diseases, suggesting that lncRNAs may become new targets for disease diagnosis and treatment (6). More importantly, the abnormal expression of lncRNAs is related to the metastasis of many malignant tumors. It has been proved that HOX transcript antisense intergenic RNA (HOTAIR), a 2.2 kb-long lncRNA transcribed from the HOXC locus, plays a role in various diseases, such as cancers (7, 8), cardiovascular diseases (9), and rheumatoid arthritis (10). The expression of HOTAIR was analyzed in breast cancer samples and discovered that the high expression of HOTAIR facilitates metastasis and death (11). In breast cancer cell lines, the overexpression of HOTAIR enhances the cell colony formation, while its down-regulation reduces invasiveness of cancer cells in vitro. The abnormal expression of HOTAIR has a certain impact on cell proliferation and apoptosis. Through silencing HOTAIR the expressions of autophagy-related genes are down-regulated in oral squamous cell carcinoma (OSCC), autophagy is inhibited, the apoptosis rate rises, and the proliferation, migration and invasion of OSCC cells are suppressed (12). Moreover, in recent years, the overexpression of HOTAIR can promote the occurrence and metastasis of breast cancer, colorectal cancer and ovarian cancer, whereas the proliferation and invasion ability of cancer cells can be effectively inhibited in the case of HOTAIR knockout (13–15). However, the special function of HOTAIR in the progression of melanoma and its mechanism remain to be further studied.

The molecular mechanism of melanoma metastasis has not been fully determined. Recent studies have found that the HOX gene plays an important role in this process, and it is regulated by specific ncRNAs, some of which are present at the HOX locus. In particular, the gene behind the HOXC locus where HOTAIR is located has abnormal expression during the development of skin melanoma (16). As an important transcription factor, nuclear factor-ϰB (NF-ϰB) regulates various cellular signaling pathways, and participates in cell death, nuclear proliferation and cell proliferation (17). The role of NF-ϰB is to promote the growth of cancer cells, and create a microenvironment suitable for proliferation of cancer cells without being rejected by the immune system (18).

In this study, the effects of small-interfering (si)-HOTAIR transfection on the proliferation and apoptosis of malignant melanoma cells were detected via cell counting kit-8 (CCK-8) assay, 4’,6-diamidino-2-phenylindole (DAPI) staining and flow cytometry, respectively, the changes in NF-ϰB pathway-related proteins and apoptosis-associated proteins after interference in lncRNA HOTAIR were detected via quantitative reverse transcription-polymerase chain reaction (qRTPCR) and Western blotting, and the regulatory mechanism of the NF-ϰB pathway was further explored.

## Materials and Methods

### Main experimental materials and instruments

Malignant melanoma A375 cells were purchased from the American Type Culture Collection (ATCC) (Manassas, VA, USA). HOTAIR siRNA (RiboBio, Guangzhou, China), Dulbecco’s modified Eagle medium (DMEM) (Gibco, Rockville, MD, USA), fetal bovine serum (FBS) (Gibco, Rockville, MD, USA), TRIzol lysis buffer (Ambion, Austin, TX, USA), SYBR Green reverse transcription kit (TaKaRa, Tokyo, Japan), cell counting kit-8 (CCK-8) cell proliferation assay kit (Beyotime Biotechnology, Shanghai, China), 4’,6-diamidino-2-phenylindole (DAPI) (J&K Chemicals), electrochemiluminescence (ECL) kit (Thermo Scientific, Waltham, MA, USA), polyvinylidene fluoride (PVDF) membrane (Millipore, Billerica, MA, USA), mouse anti-human B-cell lymphoma-2 (Bcl-2) and Bcl-2 associated X protein (Bax) primary antibodies (Santa Cruz, Santa Cruz, CA, USA), goat anti-rabbit secondary antibodies (EpigenTek, Farmingdale, NY, USA), proteinase K (Nanjing SunShine Biotechnology Co., Ltd., Nanjing, China), Bradford protein concentration assay kit (Thermo Scientific, Waltham, MA, USA), NF-ϰB activity assay kit (Cayman, Ann Arbor, MI, USA), inverted fluorescence microscope (Olympus, Tokyo, Japan), CO_2_ incubator, nucleic acid quantitative analyzer and multi-functional microplate reader (Thermo Scientific, Waltham, MA, USA), fluorescence qPCR instrument (ABI, Applied Biosystems, Foster City, CA, USA), flow cytometer (Becton Dicknson Bioscience, Franklin Lakes, NJ, USA), protein electrophoresis instrument and gel imaging system (Bio-Rad, Hercules, CA, USA).

### Cell culture and transfection

After resuscitation, the cells were subcultured in DMEM containing 10% FBS in an incubator with 5% CO_2_ at 37°C. A375 cells in the logarithmic growth phase were inoculated into a 6-well plate, and 2 mL of culture solution was added into each well. When 50–70% of cells were fused after about 24 h, they were transfected with si-HOTAIR as HOTAIR transfection (si-HOTAIR) group and empty vectors as negative control (si-NC) group according to the instructions of the transfection kit, while those without any treatment were used as blank control (control) group. After transfection, the cells were incubated in the CO_2_ incubator, and then collected for subsequent experiments.

### Detection of cell proliferation via CCK-8 assay

After trypsin digestion, A375 cells were collected *via* centrifugation, and inoculated into a 96-well plate (2000 cells/well), with three replicates in each group. After culture in DMEM containing 15% FBS for a certain period of time, the cell activity was detected using the CCK-8 kit according to the instructions. Finally, the optical density at 450 nm (OD450 nm) was detected in each group using a microplate reader, and the proliferation curve was plotted, based on which the cell proliferation ability was evaluated.

### DAPI staining assay

After transfection, A375 cells were fixed with paraformaldehyde for 5 min, and washed twice with phosphate-buffered saline (PBS), followed by DAPI staining at room temperature for 10 min. After washing twice with PBS, the apoptosis status was observed under a microscope.

### Detection of apoptosis via flow cytometry

After transfection, A375 cells were digested with an appropriate amount of 0.25% trypsin, centrifuged, collected and fixed with 70% absolute ethanol at room temperature for 5 min. Then an appropriate number of cells were resuspended in PBS, washed twice and centrifuged. After the supernatant was discarded, the cells were added and mixed evenly with Annexin V-FITC binding buffer, and incubated at room temperature for 15 min in the dark, followed by detection using the flow cytometer.

### Determination of gene expression using qRT-PCR

The cells in the logarithmic growth phase were collected and added with TRIzol reagent to extract the total RNA, and the purity and concentration of RNA were detected using the nucleic acid quantitative analyzer. Then the RNA was reversely transcribed into cDNA, followed by PCR amplification using a 20 μL of system (2 μL of cDNA, 10 μL of mix, 2 μL of primers, and 6 μL of ddH_2_O) under the following reaction conditions: pre-denaturation at 94 °C for 3 min, denaturation at 94 °C for 30 s, annealing at 55 °C for 30 s, and extension at 72 °C for 45 s, a total of 35 cycles.

The primer sequences of target genes and internal reference GAPDH were designed according to GenBank and synthesized by Beijing TsingKe Biological Technology Co., Ltd. (Beijing, China) (Table 1). The expressions of target genes were detected *via* qRT-PCR. The relative expressions of genes were statistically analyzed using the 2^−ΔΔCt^ method.

**Table 1:** PCR primers used in the study

***MRNA***	***Sequence***
HOTAIR	F: 5’-GGCAAATGTCAGAGGGTT-3’
	R: 5’-GTGTAACAGGCAGGTGGA-3’
Bcl-2	F: 5’-CGACTTCTTCAGCATCAGGA-3’
	R: 5’-TGAGCCACAGGGAGGTTCT-3’
Bax	F: 5’-ATGCGTCCACCAAGAAGC-3’
	R: 5’-CAGTTGAAGTTGCCATCAGC-3’
P65	F: 5’-GACCTGGAGCAAGCCATTAG-3’
	R: 5’-CACTGTCACCTGGAAGCAGA-3’
GAPDH	F: 5’-ACCACAGTCCATGCCATCAC-3’
	R: 5’-TCCACCACCCT GTTGCTGTA-3’

### Determination of protein expression using Western blotting

The cells in each group were washed with PBS for 3 times on ice, and fully lysed with an appropriate amount of lysis buffer prepared proportionally to release the cell protein. After centrifugation, the supernatant was collected and the protein concentration in each group was determined. The protein gel was prepared according to the molecular weight of the protein. After loading and electrophoresis, the sodium dodecyl sulphate-polyacrylamide gel electrophoresis (SDS-PAGE) gel containing the target protein was cut. Then the protein was transferred onto a PVDF membrane, sealed, and incubated with primary antibodies overnight and with secondary antibodies again for 1 h. After the membrane was evenly applied with developing solution, the protein band was scanned and quantified using a membrane scanner. Finally, Western blotting bands were quantified using Image Lab software, and the expression levels of corresponding proteins in each group were calculated.

### Detection of NF-ϰB activity via ELISA

The nuclear protein was extracted in each group and added into the 96-well plate, with 3 replicates in each group. Then the activity of NF-ϰB was detected using the NF-ϰB p65 assay kit according to the instructions. Finally, the absorbance at 450 nm was measured using the microplate reader in each group.

### Statistical analysis

The data were expressed as mean ± standard error, and analyzed using SPSS 20.0 (IBM, Armonk, NY, USA) combined with GraphPad Prism software (Version X; La Jolla, CA, USA). *P*<0.05 suggested the statistically significant difference.

## Results

### Expression of lncRNA HOTAIR in each group

**Fig. 1:**
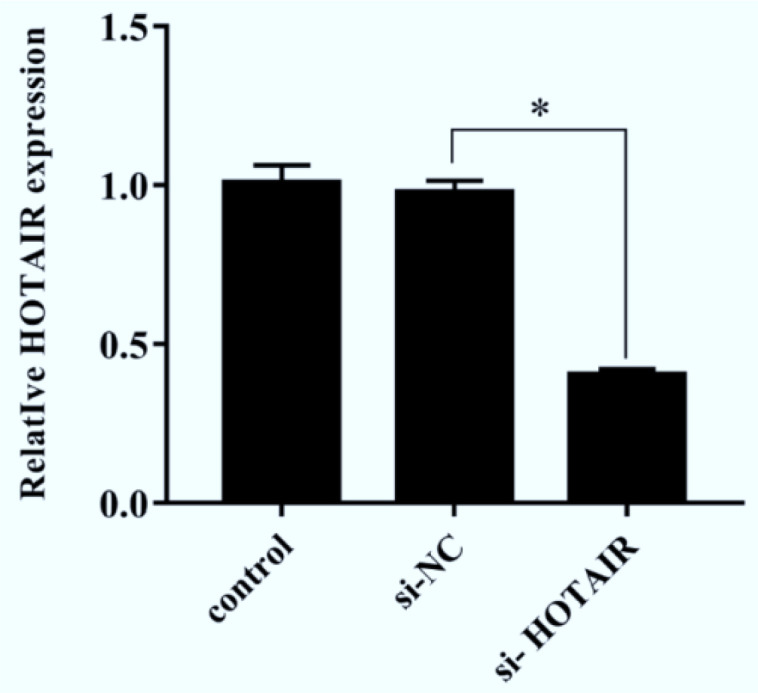
Expression of lncRNA HOTAIR in A375 cells in each group. Transfection with si-HOTAIR can reduce the HOTAIR expression in malignant melanoma cells. (^*^P<0.05)

The total RNA was extracted from cells in each group, and the expression of lncRNA HOTAIR in each group was detected *via* qRT-PCR. The results revealed that the expression of lncRNA HOTAIR in si-HOTAIR group was significantly down-regulated compared with that in si-NC group (*P*<0.05, Fig. 1), indicating the optimal transfection efficiency, and the transfection with si-HOTAIR can reduce the HOTAIR expression in malignant melanoma A375 cells.

### Effect of si-HOTAIR transfection on cell proliferation

**Fig. 2:**
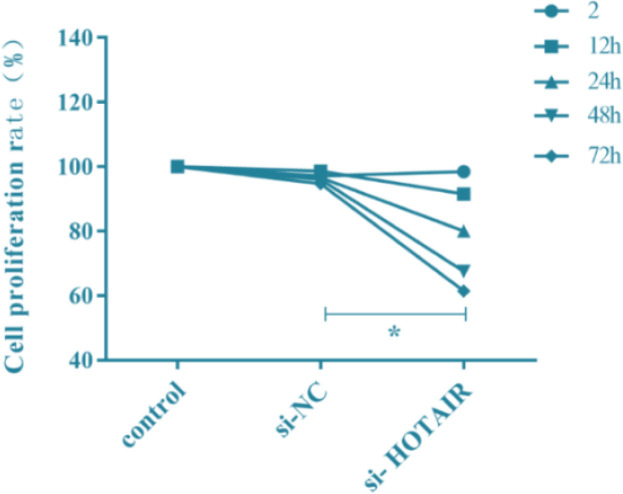
Effect of si-HOTAIR transfection on proliferation of malignant melanoma cells. Down-regulation of HOTAIR can lower the proliferation rate of A375 cells. (^*^P<0.05)

The effect of lncRNA HOTAIR on cell proliferation was determined using the CCK-8 assay, and the proliferation rate of A375 cells was detected in each group at 2 h, 12 h, 24 h, 48 h and 72 h. The results showed that the cell proliferation rate in si-HOTAIR group significantly declined gradually in si-HOTAIR group compared with that in si-NC group and control group (normalized to be 100%) (*P*<0.05) (Fig. 2), suggesting that down-regulation of HOTAIR can lower the proliferation rate of A375 cells.

### Effect of si-HOTAIR transfection on cell growth

**Fig. 3:**
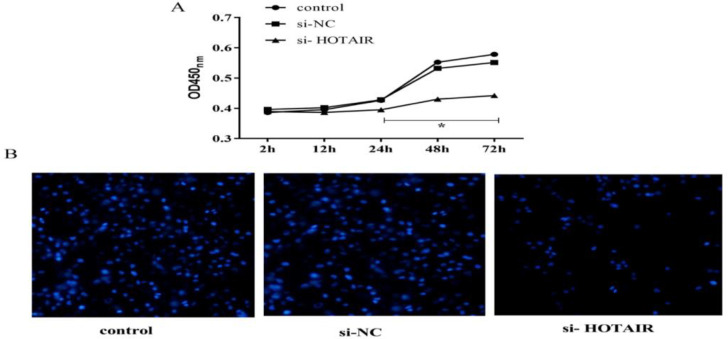
Transfection with si-HOTAIR reduces the growth of malignant melanoma cells. A, The results of CCK-8 assay show that the cell growth in si-HOTAIR group is evidently inhibited. B, The results of DAPI staining show that the number of cells in si-HOTAIR group is obviously reduced. (magnification: 400×) (^*^P<0.05)

To study the effect of si-HOTAIR transfection on the growth of malignant melanoma cells, the changes in cell growth were detected *via* CCK-8 assay and DAPI staining assay in each group. It was found that there was no obvious difference in cell growth among the three groups within 24 h, and the OD_450 nm_ was obviously lower in si-HOTAIR group than that in si-NC group and control group at 48 h and 72 h (*P*<0.05) (Fig. 3A). It was confirmed via DAPI staining that the morphology and number of malignant melanoma cells had no obvious differences between si-NC group and control group, and the number of cells in si-HOTAIR group was obviously reduced (*P*<0.05) (Fig. 3B), indicating that transfection with si-HOTAIR can inhibit the growth of malignant melanoma cells.

### Effect of si-HOTAIR transfection on apoptosis

**Fig. 4:**
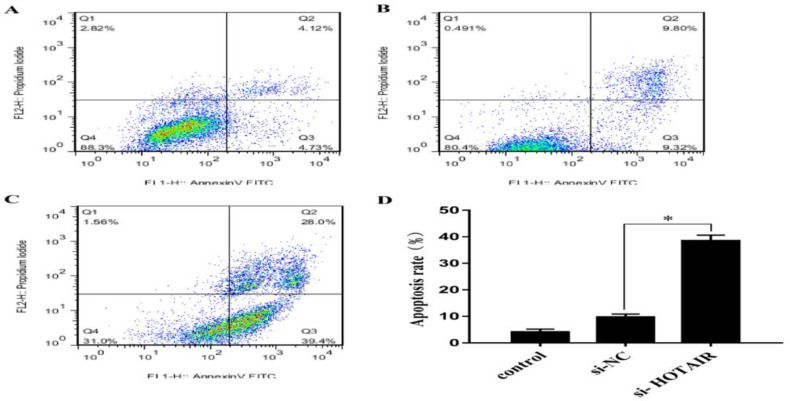
Effect of si-HOTAIR transfection on apoptosis. A–C, Apoptosis of malignant melanoma cells detected *via* flow cytometry in control group, si-NC group and si-HOTAIR group. D, Comparison of apoptosis rate of malignant melanoma cells in each group. Down-regulation of lncRNA HOTAIR can suppress the proliferation of malignant melanoma cells. (^*^P<0.05)

To explore whether down-regulation of lncRNA HOTAIR promotes the apoptosis of malignant melanoma cells, flow cytometry was performed in each group after transfection. As shown in Fig. 4, the early apoptosis rate in si-HOTAIR group (39.4%, Fig. 4C) was evidently higher than that in si-NC group (9.32%, Fig. 4B) and control group (4.73%, Fig. 4A) (*P*<0.05), confirming that down-regulation of lncRNA HOTAIR can suppress the proliferation and promote the apoptosis of malignant melanoma cells (Fig. 4D).

### Effect of down-regulation of HOTAIR on NF-ϰB signaling pathway

**Fig. 5:**
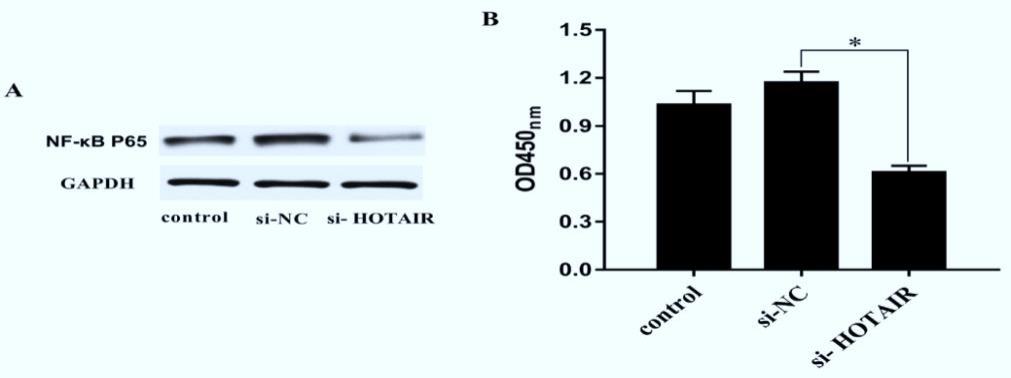
Effect of down-regulation of HOTAIR on NF-ϰB signaling pathway. ***A,*** Western blotting bands of NF-ϰB pathway proteins in control group, si-NC group and si-HOTAIR group. ***B,*** Comparison of OD of NF-ϰB pathway proteins in each group. Down-regulation of HOTAIR can inhibit the activity of NF-ϰB and promote the apoptosis. (^*^P<0.05)

To explore the effect of down-regulation of HOTAIR on the NF-ϰB signaling pathway, the expression of NF-ϰB P65 protein and the activity of NF-ϰB were detected using Western blotting and ELISA, respectively. The results manifested that after down-regulation of HOTAIR, the expression of NF-ϰB P65 protein declined, and the activity of NF-ϰB was weakened in si-HOTAIR group compared with that in si-NC group and control group (*P*<0.05) (Fig. 5), demonstrating that down-regulation of HOTAIR can inhibit the activity of NF-ϰB and promote the apoptosis.

### Effects of down-regulation of HOTAIR on expressions of apoptosis-related genes and proteins in malignant melanoma cells

**Fig. 6:**
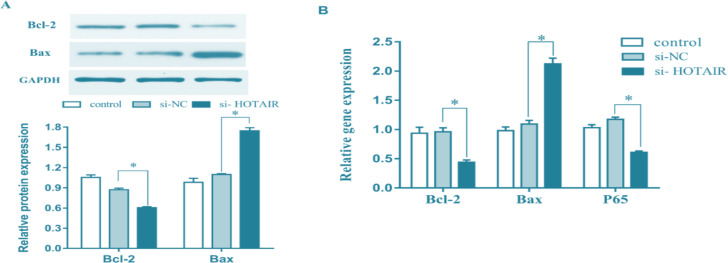
HOTAIR regulates the expressions of apoptosis-related genes and proteins. ***A,*** Expressions of apoptosis-related proteins detected using Western blotting. ***B,*** Expressions of apoptosis-related genes detected using qRTPCR. Down-regulation of HOTAIR can remarkably raise the gene and protein expressions of pro-apoptotic Bax, and remarkably lower the gene and protein expressions of anti-apoptotic Bcl-2. (^*^P<0.05)

The effects of down-regulation of HOTAIR on the expressions of apoptosis-related genes and proteins in malignant melanoma cells were detected using qRT-PCR and Western blotting. As shown in Fig. 6, compared with si-NC group and control group, si-HOTAIR group had remarkably increased gene and protein expressions of pro-apoptotic Bax, and remarkably decreased gene and protein expressions of anti-apoptotic Bcl-2 (*P*<0.05), demonstrating that the promoting effect of HOTAIR down-regulation on apoptosis may be related to the regulation on expressions of apoptosis-related genes and proteins.

## Discussion

Melanoma is one of skin cancers caused by a variety of factors, and it can be easily mistaken for benign skin disease. However, melanoma has the risk of rapid proliferation and proneness to metastasis (19). In recent years, lncRNAs play multiple roles in a variety of biological activities, including proliferation, apoptosis and inflammation (20). NcRNAs can regulate autophagy pathways and effector molecules at different autophagy stages in many types of cancers (21). HOTAIR, the first gene discovered to have cis- and trans-regulatory effects on gene expression, is overex-pressed in many tumors, such as breast cancer, colorectal cancer and oral cancer, which is closely related to the tumor progression, metastasis and prognosis of patients (11, 13, 22). The expression of HOTAIR is obviously up-regulated in primary and metastatic breast cancer compared with that in normal breast tissues (11). Moreover, HOTAIR facilitates the growth and metastasis of melanoma cells via competitively binding to miR-152-3p and activating the downstream PI3K/Akt/mTOR signaling pathway (23). It can be seen that HOTAIR can promote the occur-rence and metastasis of many cancers.

To confirm whether down-regulation of HOTAIR can inhibit the growth of melanoma cells, lncRNA HOTAIR siRNAs were designed and synthesized, and the effects of HOTAIR down-regulation on the proliferation and apoptosis of malignant melanoma cells were determined using CCK-8 assay and DAPI staining assay. Transfection with si-HOTAIR could reduce the expression of HOTAIR in malignant melanoma cells. The results of CCK-8 assay revealed that the cell proliferation rate significantly declined gradually in si-HOTAIR group compared with that in si-NC group and control group. It was confirmed via CCK-8 assay and DAPI staining assay that the number of cells in si-HOTAIR group was obviously reduced, which indicates that down-regulation of HOTAIR can reduce the proliferation of malignant melanoma cells.

Through silencing HOTAIR the expression of autophagy-related genes in OSCC is down-regulated, the autophagy is suppressed and the apoptosis rate rises (12). In this study, to explore whether down-regulation of lncRNA HOTAIR promotes the apoptosis of malignant melanoma cells, flow cytometry was performed in each group after transfection. The evidently higher early apoptosis rate was observed after down-regulation of HOTAIR, confirming that down-regulation of lncRNA HOTAIR can suppress the proliferation and promote the apoptosis of malignant melanoma cells.

NF-ϰB can stimulate the expressions of a variety of cytokines and chemokines, and participate in epithelial cell growth and activity (24, 25). The role of NF-ϰB is to promote the growth of cancer cells. It is speculated that NF-ϰB is involved in the development of malignant melanoma. In this study, the expression of NF-ϰB P65 protein and the activity of NF-ϰB were detected using Western blotting and ELISA, respectively. The results manifested that after down-regulation of HOTAIR, the expression of NF-ϰB P65 protein declined, and the activity of NF-ϰB was weakened, demonstrating that down-regulation of HOTAIR can inhibit the activity of NF-ϰB. In addition, the effects of down-regulation of HOTAIR on the expressions of apoptosis-related genes and proteins in malignant melanoma cells were detected using qRT-PCR and Western blotting, respectively.

The results manifested that down-regulation of HOTAIR could remarkably raise the gene and protein expressions of pro-apoptotic Bax, and remarkably lower the gene and protein expressions of anti-apoptotic Bcl-2, indicating that the promoting effect of HOTAIR down-regulation on apoptosis may be related to the regulation on expressions of apoptosis-related genes and proteins.

## Conclusion

Down-regulation of lncRNA HOTAIR can inhibit the proliferation and promote apoptosis of malignant melanoma cells and suppress the NF-ϰB pathway, which may be related to the regulation on the NF-ϰB signaling pathway. The research results suggest that HOTAIR may become a potential therapeutic target for malignant melanoma.
